# Advances in CO_2_ Laser Treatment of Cotton-Based Textiles: Processing Science and Functional Applications

**DOI:** 10.3390/polym18020193

**Published:** 2026-01-10

**Authors:** Andris Skromulis, Lyubomir Lazov, Inga Lasenko, Svetlana Sokolova, Sandra Vasilevska, Jaymin Vrajlal Sanchaniya

**Affiliations:** 1 Engineering Center, Riga Technical University—Rezekne Academy, Atbrīvošanas Aleja 115, LV-4601 Rezekne, Latvia; 2 Institute of Mechanical and Biomedical Engineering, Riga Technical University, Ķīpsalas iela 6A, LV-1048 Rīga, Latvia

**Keywords:** CO_2_ laser finishing, cotton textiles, denim, photothermal interaction, oxidation, micro-ablation, laser functionalisation, sustainable textile processing, Industry 4.0

## Abstract

CO_2_ laser processing has emerged as an efficient dry-finishing technique capable of inducing controlled chemical and morphological transformations in cotton and denim textiles. The strong mid-infrared absorption of cellulose enables localised photothermal heating, leading to selective dye decomposition, surface oxidation, and micro-scale ablation while largely preserving the bulk fabric structure. These laser-driven mechanisms modify colour, surface chemistry, and topography in a predictable, parameter-dependent manner. Low-fluence conditions predominantly produce uniform fading through fragmentation and oxidation of indigo dye; in comparison, moderate thermal loads promote the formation of carbonyl and carboxyl groups that increase surface energy and enhance wettability. Higher fluence regimes generate micro-textured regions with increased roughness and anchoring capacity, enabling improved adhesion of dyes, coatings, and nanoparticles. Compared with conventional wet processes, CO_2_ laser treatment eliminates chemical effluents, strongly reduces water consumption and supports digitally controlled, Industry 4.0-compatible manufacturing workflows. Despite its advantages, challenges remain in standardising processing parameters, quantifying oxidation depth, modelling thermal behaviour, and assessing the long-term stability of functionalised surfaces under real usage conditions. In this review, we consolidate current knowledge on the mechanistic pathways, processing windows, and functional potential of CO_2_ laser-modified cotton substrates. By integrating findings from recent studies and identifying critical research gaps, the review supports the development of predictable, scalable, and sustainable laser-based cotton textile processing technologies.

## 1. Introduction

The introduction of CO_2_ laser systems into textile finishing has transformed the way in which cotton and denim fabrics are faded, patterned, and functionally modified. The fundamental driver of this transformation is the strong absorption of 10.6 μm radiation by cellulose, which enables highly localised photothermal interaction within the near-surface region of the fibres. Under well-controlled conditions, this interaction makes it possible to adjust colour, surface chemistry, and morphology without compromising the bulk mechanical integrity of the fabric, thereby offering a precise, digitally controllable alternative to traditional wet processing [1,2,3].

Conventional denim finishing methods, such as hypochlorite bleaching, permanganate spraying, enzyme washing and mechanical abrasion, rely on large volumes of water, multiple chemical baths and intensive washing and drying cycles. These processes generate substantial amounts of chemical effluent and impose significant environmental and regulatory burdens on textile mills [4,5,6,7]. In response to increasing ecological and legislative pressure, there has been a pronounced shift towards clean and resource-efficient technologies, among which CO_2_ laser processing is one of the most mature and widely implemented solutions for denim and cotton finishing [3,8].

Industrial adoption has outpaced the development of a unified scientific framework. Different studies report successful fading, patterning, and surface activation under widely differing combinations of fluence, scanning speed, resolution, spot size, and focusing conditions, often with limited documentation of experimental parameters [9,10,11,12,13]. While the macroscopic outcomes, such as shade change and pattern sharpness, are routinely reported, the underlying sequence of photothermal, oxidative, and ablative mechanisms is still not fully standardised. As a result, predictive processing windows that link specific parameter ranges to well-defined surface states are only partially established.

Concurrently, there is growing evidence that CO_2_ laser treatment provides far more than mere aesthetic fading. Localised oxidation and micro-texturing can significantly increase surface polarity, wettability, and anchoring capacity, thereby enabling improved dyeability, stronger adhesion of hydrophobic, antimicrobial and conductive coatings and efficient immobilisation of metallic and metal-oxide nanoparticles [14,15,16,17]. As a result, CO_2_ laser processing is evolving from a decorative technology into a platform for functional, performance-driven textile engineering [18].

The aim of this review is to provide a mechanistically grounded and critically structured analysis of CO_2_ laser interaction with cotton and denim textiles. The discussion begins with the photothermal fundamentals of laser–cellulose interaction, proceeds through the role of processing parameters and material factors, examines the resulting structural, chemical and mechanical transformations and then addresses post-laser functionalisation, sustainability aspects and industrial integration. Lastly, current research gaps and future directions are analysed with a view towards standardised, data-driven and scalable laser-based textile technologies.

To address the recent acceleration of research in digital and sustainable textile manufacturing, we screened the literature with an emphasis on work published from 2019 to 2025, while retaining earlier seminal mechanistic studies that established the photothermal interaction framework. The scope is restricted to CO_2_ laser processing of cotton-based textiles (pure cotton, denim, and cotton-rich blends where cotton/cellulose is the primary interacting constituent) and to outcomes that can be directly linked to CO_2_-laser-induced photothermal heating, dye decomposition, cellulose oxidation, and micro-ablation, as well as to post-laser functionalisation enabled by these surface changes.

The search strategy targeted peer-reviewed, English-language publications indexed in major scholarly databases and was complemented by keyword-based screening of widely used academic search engines. Query strings combined terms for the laser source (“CO_2_ laser”, “10.6 µm”), substrate (“cotton”, “cellulose”, “denim”), and application or mechanism (“fading”, “surface activation”, “oxidation”, “ablation”, “functionalisation”). Records were screened for direct relevance to CO_2_ laser processing of cotton-based substrates and for the presence of adequately described process variables (e.g., power/fluence, scanning speed, resolution/overlap, beam diameter/focus) and measurable responses (e.g., colour metrics, surface chemistry, morphology, wettability, mechanical properties, or durability).

Emerging hybrid routes (laser combined with plasma, ultrasound, or other physical pre-treatments) and emerging material classes (regenerated cotton and functional blended cotton, such as cotton/linen or cotton/recycled polyester) were also screened. However, within the CO_2_ laser + cotton focus and within the indexed corpus available to the authors at the time of preparation, these hybrid and next-generation blend studies were not sufficiently represented to support a systematic comparative collation at the same maturity level as the conventional cotton and denim literature. We, therefore, treat these topics as research gaps and specify, where relevant, the inclusion boundaries and the resulting limitations of the present review.

No geographic or institutional restrictions were applied during screening. Nevertheless, some residual bias is possible due to database coverage, language filtering, and non-uniform reporting of process parameters. These limitations further motivate the need for standardised reporting and open, cross-laboratory datasets in this field.

## 2. Mechanistic Fundamentals of CO_2_ Laser–Cotton Interaction

### 2.1. Photothermal Energy Deposition in Cellulose

The interaction between CO_2_ laser radiation and cotton is dominated by photothermal mechanisms. Cellulose exhibits strong absorption in the mid-infrared region around 10.6 μm due to vibrational excitation of C–O, C–H and O–H bonds. When a cotton surface is irradiated by a CO_2_ laser, the absorbed energy is rapidly converted into heat within the outer fibre layers, giving rise to steep temperature gradients that evolve on millisecond time scales [19,20,21,22]. Because the wavelength is non-ionising and the pulse durations in common industrial systems are orders of magnitude longer than those used in ultrafast processing, non-thermal and plasma-related mechanisms can be disregarded, and the interaction can be treated as purely thermal.

The spatial and temporal distribution of temperature is determined by the incident fluence, the dwell time per pixel, the spot size and the thermal diffusivity of the fabric. Thermal diffusion within the woven or knitted structure is anisotropic and depends on yarn geometry and fabric density, which explains the sometimes significant differences in laser response between plain weave cotton, twill denim, and cotton/polyester blends [19,23]. At sufficiently low energy densities, the photothermal field remains confined to the uppermost fibre shells, limiting the extent of chemical and morphological modification.

### 2.2. Thermal Decomposition of Indigo Dye

On indigo-dyed denim, the most apparent and technologically exploited effect of CO_2_ laser treatment is visual fading. Indigo is thermally and oxidatively labile. Localised heating disrupts its conjugated chromophore, leading to bond cleavage, fragmentation and oxidation of dye molecules located at or near the fibre surface [24,25]. Based on the delivered thermal load, the process can progress from partial decolouration, through uniform shade reduction, to almost complete removal of pigment in the exposed zones.

The results of spectroscopic and colourimetric studies have shown that under moderate exposure conditions, indigo decomposition is essentially a near-surface phenomenon, and the underlying cellulose structure remains largely intact [26,27,28]. At higher fluence, indigo may partially volatilise or sublime, and the removal of surface pigment can be accompanied by oxidative modification of the fibre itself. Indigo decomposition thus forms the first step in a mechanistic sequence that, under more severe conditions, transitions into cellulose oxidation and, eventually, micro-ablation.

### 2.3. Oxidation of Cellulose and Formation of Functional Groups

Once the surface temperature exceeds the onset of thermal degradation, cellulose begins to undergo dehydration and oxidation reactions. These processes generate carbonyl, carboxyl, and related oxygen-containing functional groups at the fibre surface [6,10,12,29,30,31]. The formation of such groups increases surface polarity and wettability, which can be observed as a reduction in water contact angle and an enhancement of capillary uptake.

FTIR spectra of CO_2_ laser-treated cotton fabrics consistently show an increase in absorption bands associated with oxidised cellulose, particularly:

- ~1730–1745 cm^−1^, corresponding to carbonyl (C=O) stretching vibrations,
- ~1240–1260 cm^−1^, attributed to carboxyl (O–C=O) groups,
- and changes in the ~1600–1650 cm^−1^ region related to oxidised cellulose structures [18].

These spectral changes are indicative of photothermally induced oxidation of cellulose, where primary hydroxyl groups are partially converted into aldehyde, ketone, and carboxylic functionalities. Importantly, this oxidation is largely confined to the fibre surface (micrometre scale), while the bulk cellulose structure remains intact under optimised processing conditions. The increase in oxygen-containing functional groups explains the experimentally observed enhancement in surface polarity, wettability, dyeability and coating adhesion after CO_2_ laser treatment [18,32].

X-ray photoelectron spectroscopy measurements further corroborate the FTIR findings by demonstrating a noticeable increase in the O/C atomic ratio at the fibre surface, and the emergence and growth of characteristic C1s components at:

- ~286.5 eV (C–O),
- ~288.0 eV (C=O),
- ~289.0 eV (O–C=O).

These results confirm that CO_2_ laser irradiation acts as a dry, spatially selective oxidative surface modification technique, generating chemically active functional groups that are highly relevant for subsequent dyeing, coating and functionalization processes [18,33]. The depth of the oxidised layer is typically confined to a few micrometres, yet this depth is sufficient to profoundly influence interfacial interactions with aqueous dyes, coating precursors and nanoparticle suspensions. The laser effectively acts as a localised oxidative pretreatment, eliminating the need for wet chemical activation in many cases.

### 2.4. Micro-Ablation and Morphological Evolution

At higher local temperatures and longer dwell times, thermal stresses within the fibre surface lead to micro-scale ablation, which can take the form of fibril detachment, shallow pits, micro-cracks and general surface roughening [9,18,32]. SEM images of CO_2_ laser-treated cotton fabrics consistently demonstrate a fluence-dependent morphological progression:

- Low fluence: Fibres retain relatively smooth surfaces with intact cuticle layers. Morphological changes are minimal, and the dominant mechanism is indigo dye decomposition, resulting in visual fading with negligible fibre damage.
- Moderate fluence: Partial oxidation of cellulose leads to micro-fibrillation and moderate surface roughening. The increased surface area and roughness enhance interfacial interactions, making this regime optimal for functional activation without significant mechanical degradation.
- High fluence: Pronounced micro-ablation, including pits, cracks and exposure of inner fibre structures, is observed. At this stage, thermal damage and partial carbonisation may occur, which negatively affects mechanical integrity [9,18,32].

This morphological evolution provides direct visual evidence for the transition from fading-dominated mechanisms to oxidation-driven surface activation and, finally, to destructive ablation at excessive thermal loads.

The micro-scale features mentioned above increase the specific surface area and can improve mechanical interlocking with subsequently applied coatings or polymer layers. In denim fabrics, micro-ablation often follows the yarn structure, creating pseudo-relief patterns that contribute to the characteristic worn appearance associated with laser finishing. Concurrently, excessive ablation can expose inner fibre regions and reduce tensile strength and abrasion resistance, highlighting the need for careful parameter control [33,34].

### 2.5. Mechanistic Progression and Processing Regimes

The combined photothermal mechanisms follow a characteristic progression as the local thermal budget increases. At low fluence and short exposure times, indigo dye decomposition dominates, producing uniform fading with minimal fibre modification [35]. At intermediate energy densities, dye removal is accompanied by oxidation of cellulose and the generation of polar functional groups, resulting in a chemically activated surface with enhanced wettability and reactivity [36,37]. At still higher fluence and longer dwell times, micro-ablation and more pronounced morphological changes occur, increasing roughness and anchoring capacity but also approaching the onset of structural damage [38,39]. When thermal loading exceeds critical thresholds, carbonisation and charring become evident, and the fabric loses mechanical integrity [38].

**Figure 1:**
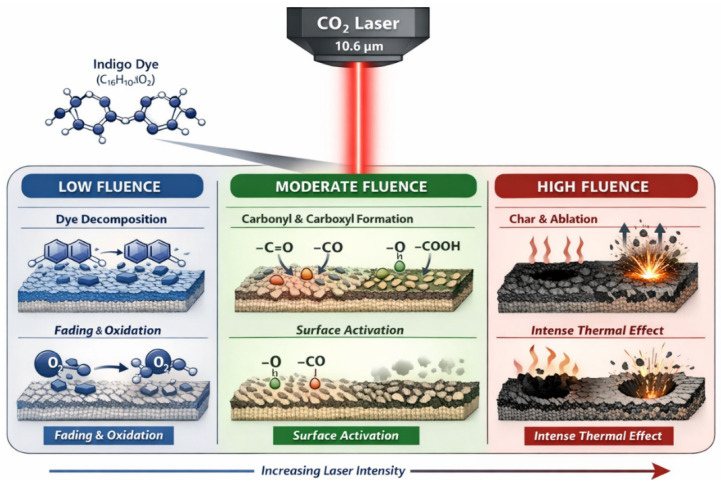
Simplified schematic of thermally driven surface transformation mechanisms during CO_2_ laser treatment of cotton: initial thermal ablation with partial removal of dye and fibre material, subsequent oxidation in the presence of oxygen and pore formation/micro-pitting on the fibre surface.

This progression can be visualised in a simplified schematic that emphasises the sequence of thermally driven surface transformations, initial thermal ablation and partial dye removal, followed by oxidation in the presence of oxygen and subsequent pore or micro-pit formation on the fibre surface, as illustrated in Figure 1. For clarity, the key phenomena shown schematically in Figure 1 and their specific interpretation in the context of CO_2_ laser interaction with cotton are summarised in Table 1.

## 3. Processing Parameters and Material Factors

**Figure 2:**
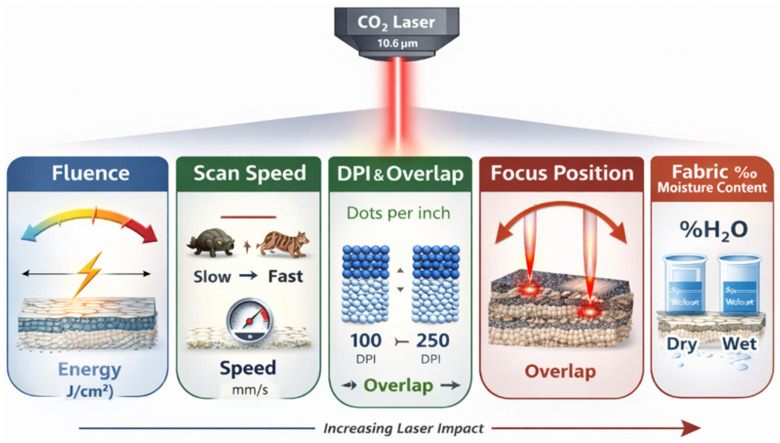
An illustrative representation of the main factor groups governing CO_2_ laser interaction with textile substrates.

The outcome of CO_2_ laser treatment is not determined by any single parameter but by the coupled action of three main factor groups: the intrinsic laser parameters (wavelength, power density and modulation), the characteristics of the technological system (scanning speed, spacing, auxiliary gas flow and optical delivery) and the thermo-physical and structural properties of the textile substrate. The laser–textile interaction process can therefore be regarded as the result of an overlap between these three domains, where any change in one group modifies the effective thermal budget experienced by the fabric and shifts the balance between fading, oxidation, micro-ablation and carbonisation. This triad of interacting factor groups is summarised schematically in Figure 2 and provides a conceptual framework for interpreting the more detailed parameter dependencies discussed in the following subsections.

Within these domains, the practical process design is usually based on a more fine-grained set of parameter groups that distinguish between laser settings, optical configuration, scanning strategy and material characteristics. A representative classification of these groups and the associated parameters is given in Table 2; in the following subsections, we discuss their influence in more detail.

### 3.1. Fluence and Effective Thermal Budget

Fluence, defined as the energy delivered per unit area, is the primary variable governing the severity of the photothermal interaction. Numerous studies demonstrate that small changes in fluence can shift the response from barely perceptible fading to severe charring [40,41,42]. At low fluence, the peak surface temperature remains below the thresholds for extensive cellulose degradation, and the process is largely limited to indigo decomposition and minor oxidation. At intermediate levels, fluence is sufficient to drive functional group formation and micro-texturing, whereas at high levels, thermal runaway and carbonisation become likely [40,41,42,43].

Fluence, however, does not act independently. For a given average power, changes in scanning speed, pulse duration and spot size will modify the effective energy density and the rate at which heat is deposited. It is, therefore, more appropriate to view fluence as one component of an overall thermal budget that includes temporal and spatial aspects of energy delivery.

### 3.2. Scanning Speed, Exposure Time and Temporal Dynamics

Scanning speed determines the residence time of the laser beam on any given point of the textile surface. Lower speeds increase the local dwell time, enabling greater heat accumulation and deeper thermal penetration, whereas higher speeds limit the peak temperature and confine the interaction to the outermost layers [12,44,45]. Experiments on denim fading consistently show that, for a fixed fluence, slower scanning produces stronger shade reduction and higher risk of yellowing or charring, while faster scanning yields softer fading and improved preservation of mechanical properties [11,12].

In systems with pulsed or pixel-based control, the exposure time per pixel interacts with repetition rate and scanning strategy. Longer pixel durations intensify local heating and can push otherwise moderate fluence into regimes where oxidation and micro-ablation become dominant. Understanding and controlling these temporal aspects is essential for defining robust processing windows, especially when transferring parameter sets between different laser platforms.

### 3.3. Resolution and Spot Overlap

Resolution, commonly expressed as dots per inch (DPI), controls the spatial density and overlap of successive laser impacts. High DPI values increase the number of spots per unit area and the degree of overlap between them, thereby raising the cumulative thermal load even if the nominal fluence per spot remains constant [46,47]. The results of optical and SEM analyses of laser-engraved denim show that increased DPI improves pattern uniformity and edge definition; however, it also increases the risk of over-processing in highly exposed regions [47,48].

In contrast, low DPI values reduce overlap and heat accumulation, which can be advantageous for soft, low contrast fading but may lead to coarse or pixelated pattern edges. The interplay between DPI, scanning speed and fluence, therefore, defines a multi-dimensional parameter space in which stable regimes for uniform fading, controlled activation and micro-texturing must be identified [47].

### 3.4. Beam Diameter, Focal Position and Optical Configuration

**Figure 3:**
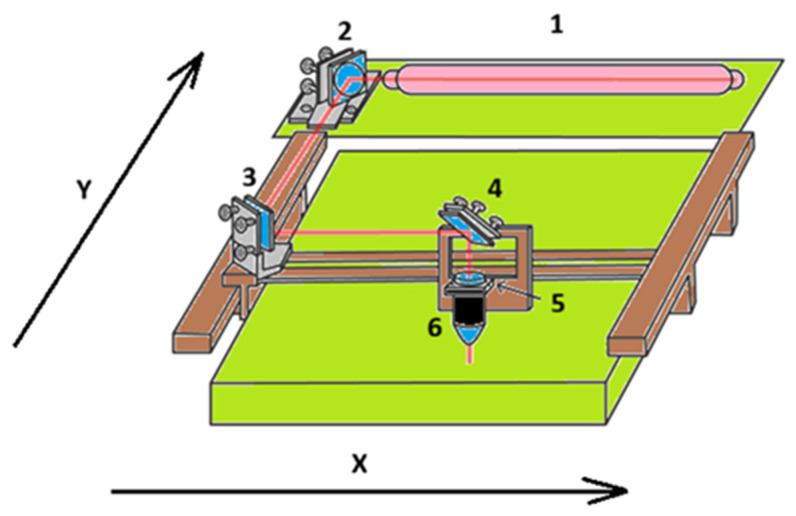
Schematic layout of a typical flatbed CO_2_ laser system for textile processing: 1—laser tube, 2—first steering mirror, 3—second steering mirror, 4—Y-direction carriage, 5—X-direction gantry, and 6—focusing head and nozzle. The laser beam is guided along the X–Y workspace and focused onto the textile surface.

In most industrial CO_2_ laser denim and cotton finishing systems, the radiation generated in the glass discharge tube is guided by plane mirrors mounted on the moving gantry and finally focused onto the textile surface by a lens located in the processing head. A simplified kinematic and optical layout of such a flatbed system is shown in Figure 3, where the beam is directed from the stationary laser source (1) via steering mirrors (2, 3) to a carriage (4, 5) that scans the focal spot along the X and Y directions over the fabric, terminating in the focusing head and nozzle (6). This configuration illustrates how laser parameters, optical elements and mechanical motion jointly define the actual beam diameter, incidence angle and stability at the workpiece.

The spatial distribution of energy on the fabric surface depends not only on fluence and scanning speed but also critically on beam diameter and focus position. A tightly focused beam concentrates energy into a small area, producing high peak intensities and strong local gradients, which favour pronounced micro-ablation, sharp pattern edges and high-resolution engraving [3,49,50]. A slightly defocused beam spreads the energy over a larger area, lowering the peak temperature and producing homogeneous, low-contrast fading.

Industrial systems typically employ galvanometric scanners or motion stages combined with f-theta lenses. Small misalignments in the beam path, lens contamination or fabric positioning can lead to spatial variation in focus and, consequently, in processing outcome across the work field. Consistent optical configuration and regular calibration are therefore prerequisites for reproducible laser finishing [48,51].

### 3.5. Fabric Composition, Structure and Moisture Content

The response of a textile to CO_2_ laser irradiation depends not only on laser settings but also on the intrinsic properties of the fabric. Pure cotton fabrics exhibit different absorption and thermal conduction behaviour compared with cotton/polyester blends, which can display local melting or shrinkage of the synthetic component [23]. However, CO_2_ laser processing is well-suited not only for marking and engraving but also for the cutting and welding of polyester fabric [52].

**Figure 4:**
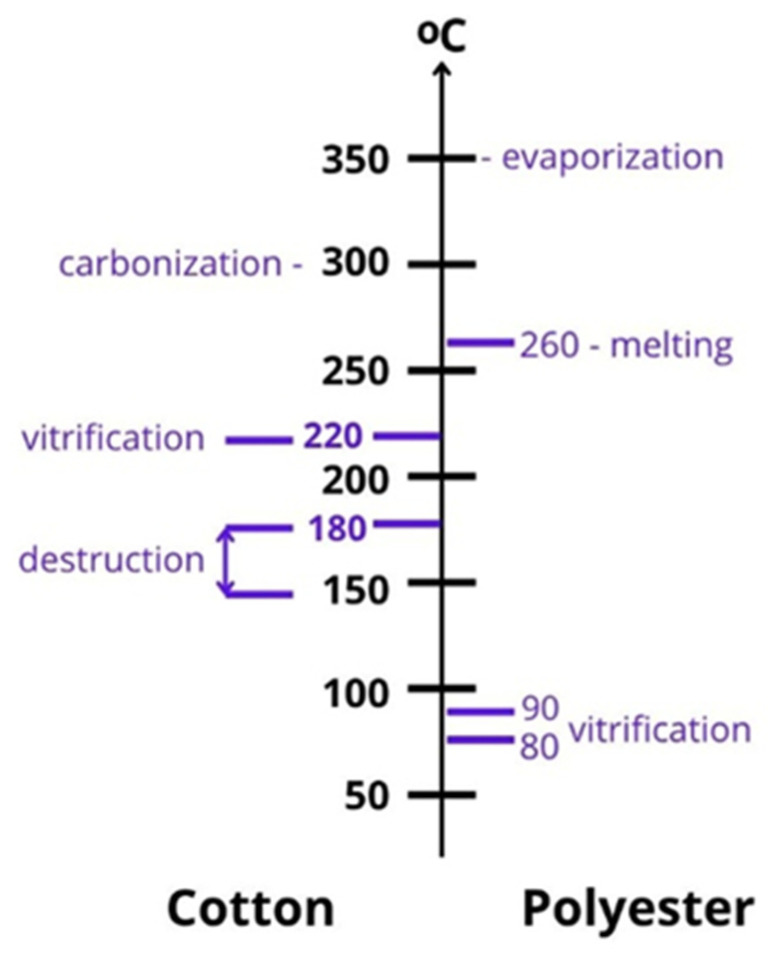
Reaction of cotton and polyester to heating when exposed to a CO_2_ laser source.

It should be noted that cotton and polyester react very differently to CO_2_ laser exposure, which is especially important when the fabric contains fibres of both of these components. See Figure 4 for more details.

Fabric density, yarn twist, weave pattern and finishing chemicals influence thermal diffusion and can affect both the uniformity of fading and the onset of micro-ablation [48].

Moisture content plays a particularly important role. The presence of water in the fibre structure increases heat capacity and can act as a thermal buffer, reducing peak temperatures and delaying the onset of oxidation and ablation [38,53]. Experiments have shown that pre-conditioning cotton fabrics at higher humidity can broaden the safe processing window and reduce the risk of over-processing at a given fluence. Conversely, dry fabrics are more susceptible to rapid temperature rise and severe local modification.

### 3.6. Parameter–Response Maps and Processing Windows

To support practical process design, it is beneficial to organise the complex interactions between fluence, scanning speed, DPI, spot size and fabric characteristics into parameter–response maps. These maps delineate regions corresponding to controlled fading, combined fading and oxidation, micro-ablation and carbonisation [48]. The authors of experimental studies on denim have constructed such maps by measuring colour difference, surface chemistry and morphology across grids of fluence and speed values [54,55]. In addition, these studies are often machine- and fabric-specific, their underlying structure is similar and provides a basis for more generalised frameworks and complements the conceptual factor grouping as illustrated in Figure 2 and the system-level configuration shown in Figure 3.

## 4. Laser-Induced Transformations of Cotton and Denim

### 4.1. Visual and Colourimetric Effects

The earliest and most directly observable consequence of CO_2_ laser treatment of indigo-dyed denim is shade modification. Colourimetric analyses typically show monotonic decreases in colour strength (K/S) and increases in lightness (L*) with increasing fluence or decreasing scanning speed [8]. At low exposure, fading is uniform and stable, and the visual effect is dominated by indigo decomposition. At intermediate exposure, more pronounced lightening is accompanied by subtle yellowing or greening, reflecting contributions from oxidised cellulose and residual dye degradation products [47].

Careful selection of parameters can enable the production of high-contrast, sharply defined patterns with minimal background damage. However, excessive exposure leads to uneven or burnt appearances, with visible carbonised spots and structural degradation. Preventing such defects requires a detailed understanding of the interplay between colour change and underlying chemical and morphological transformations.

### 4.2. Chemical Modification and Surface Activation

CO_2_ laser treatment significantly alters the surface chemistry of cotton fabrics. FTIR spectra of laser-treated samples generally exhibit an increase in bands associated with carbonyl (C=O) and carboxyl (O–C=O) groups; in comparison, XPS reveals an increased O/C ratio at the fibre surface [32]. These changes are consistent with partial oxidation of cellulose chains and the formation of functional groups such as aldehydes, ketones and carboxylic acids.

The oxidised surface exhibits higher surface energy and improved wettability, which can be quantified through contact angle measurements and capillary uptake tests [56]. Laser-induced oxidation can therefore be viewed as a dry, spatially selective alternative to conventional wet oxidative pretreatments. Importantly, the depth of oxidation is typically limited to a thin surface layer, preserving the bulk properties of the yarns while substantially modifying their interfacial behaviour.

### 4.3. Morphological Evolution and Micro-Texturing

Morphological changes induced by CO_2_ laser irradiation have been extensively characterised using SEM. Untreated cotton fibres typically appear smooth with well-defined twists and cuticle layers. After laser exposure, a progressive evolution of surface features is observed, initial fibrillation and roughening at low to moderate fluence, followed by the formation of shallow pits, micro-cracks and, in some cases, partial removal of outer layers at higher fluence [48].

These micro-scale features increase in specific surface area and can improve mechanical interlocking with subsequently applied coatings or polymer layers. In denim fabrics, micro-ablation often follows the yarn structure, creating pseudo-relief patterns that contribute to the characteristic worn appearance associated with laser finishing. Concurrently, excessive ablation can expose inner fibre regions and reduce tensile strength and abrasion resistance, highlighting the need for careful parameter control [57,58].

**Figure 5:**
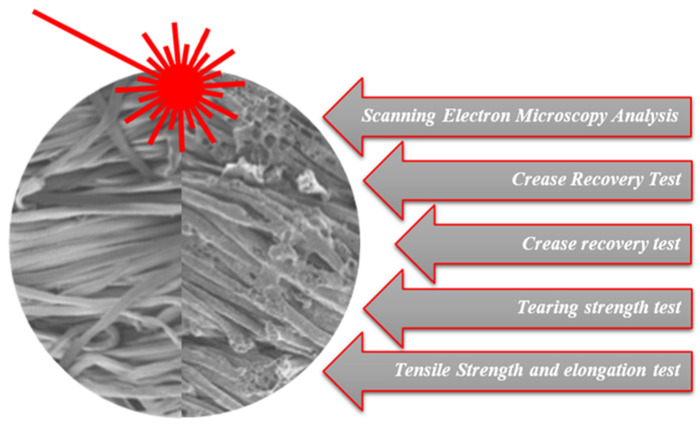
Schematic representation of the multi-scale characterisation of CO_2_ laser-treated cotton fabrics: SEM micrographs of fibres before and after laser irradiation are combined with a series of mechanical and handle tests, including scanning electron microscopy analysis, crease recovery tests, tearing strength tests and tensile strength/elongation measurements. The red arrow shows the laser beam.

From an experimental perspective, the link between microscopic morphology and macroscopic performance is typically assessed by combining SEM analysis with a suite of mechanical and handle-related tests, including crease recovery, tearing strength and tensile strength/elongation measurements. This integrated characterisation strategy is illustrated schematically in Figure 5, where SEM images of fibres before and after CO_2_ laser irradiation are coupled with the principal mechanical tests used to quantify the impact of processing on fabric performance.

Beyond detailed quantitative testing, the most frequently reported qualitative correlations between the observable macroscopic effects of CO_2_ laser processing and the dominant underlying mechanisms can be summarised in a compact form, as shown in Table 3. This overview highlights how visual appearance, surface feel and functional properties are directly linked to specific photothermal, oxidative, and ablative processes.

### 4.4. Mechanical Properties and Hand

The impact of CO_2_ laser treatment on mechanical properties depends strongly on the severity of exposure. Studies of tensile strength, elongation at break and tear resistance generally indicate that, under optimised conditions, mechanical degradation can be kept within acceptable limits, particularly when processing is confined to the fabric surface [32,59]. However, when fluence and dwell times exceed recommended ranges, significant reductions in strength and flexibility can occur due to chain scission, crosslinking and structural damage associated with carbonisation [48].

Changes in fabric hand and drape are also observed following laser processing. Subjective assessments and objective hand measurements show that excessive oxidation and ablation can increase stiffness and harshness; in comparison, moderate treatment can preserve or even slightly improve softness in some cases, particularly when it replaces aggressive mechanical or chemical finishing steps [60]. Balancing visual, chemical and mechanical outcomes is, therefore, a central challenge in process optimisation, and the combined SEM–mechanical testing approach depicted in Figure 5, together with the qualitative correlations listed in Table 3, provides a useful framework for quantifying these trade-offs.

## 5. Post-Laser Functionalisation of Cotton Surfaces

### 5.1. Enhanced Dyeability and Colour Re-Engineering

The oxidised and micro-textured surfaces produced via CO_2_ laser treatment offer improved interaction with a variety of dye classes. Reactive, direct and vat dyes all exhibit increased exhaustion and better fixation on laser-activated cotton compared with untreated substrates [61]. The combination of new functional groups and increased surface area enables higher dye uptake and more uniform penetration, with improved bonding leading to enhanced wash fastness and colour stability [47].

Laser pre-activation also facilitates localised re-dyeing or colour re-engineering of denim fabrics. Regions that have been selectively oxidised by the laser can be re-dyed without additional wet pretreatment, enabling restoration of faded areas or the creation of multicolour designs with high spatial precision [3], opening up possibilities for on-demand customisation and extended product lifetimes.

### 5.2. Hydrophobic and Barrier Coatings

The presence of polar functional groups does not preclude hydrophobic modification; on the contrary, it facilitates the covalent or strongly adsorbed attachment of hydrophobic silanes and fluorine-free water-repellent systems. Studies on laser-treated denim show that such coatings can form more uniform and durable layers on oxidised and micro-textured surfaces, with improved resistance to washing and abrasion [62,63].

By combining laser-induced micro-texturing with hydrophobic coatings, it is possible to create fabric surfaces that exhibit both high water repellence and controlled breathability. The micro-scale roughness enhances the Cassie–Baxter effect, with the underlying cotton structure maintaining vapour permeability. These features are of interest for outdoor and technical textiles where water repellence, comfort and aesthetics must be jointly optimised.

### 5.3. Antimicrobial and Biofunctional Finishes

Laser-activated cotton provides favourable conditions for the deposition and retention of antimicrobial agents, particularly those involving metal complexes or nanoparticles. The increased density of oxygen-containing functional groups enhances coordination with metal ions such as Ag^+^, Cu^2+^, and Zn^2+^, leading to stable binding of antimicrobial species at the fibre surface [64]. Experimental studies demonstrate that laser-pretreated fabrics can achieve higher and more durable antimicrobial activity compared with fabrics treated without prior activation [34].

Beyond classical antimicrobial finishes, laser-activated surfaces can also support bifunctional coatings, including those based on chitosan, peptides or other biopolymers. The improved wettability and surface energy facilitate uniform film formation, while micro-scale roughness provides additional anchoring. Such combinations enable the design of smart textiles with targeted biological interactions.

### 5.4. Conductive and Electronic Textiles

CO_2_ laser activation can contribute to the development of conductive and electronic textiles in several ways. First, it enhances adhesion and uniformity of conductive coatings based on metal nanoparticles, conductive polymers or carbon-based materials. The combination of surface polarity and micro-texturing improves the stability of such layers under flexing and laundering [65,66,67]. Second, localised laser modification can be used to define regions with distinct surface energies, guiding the placement of conductive paths or components.

In some approaches, the laser is used not only as a pretreatment tool but also as a direct patterning method for conductive inks or metallisation paths. While we focus on pretreatment and functionalisation in this review, the underlying mechanisms of surface activation remain relevant for these more advanced applications.

Representative examples include:

1. Metal nanoparticle-based conductive textiles

Laser-induced oxidation enhances the anchoring of Ag, Cu, or Ni nanoparticles through coordination with surface carboxyl and carbonyl groups. Such systems have been used for flexible conductive tracks, EMI shielding fabrics and multifunctional antibacterial–conductive textiles [68,69,70,71].

2. Laser-induced carbonaceous structures

Under higher fluence conditions, CO_2_ laser processing can generate carbon-rich or partially graphitised surface layers. These structures have been employed in strain sensors, flexible heaters and resistive sensing elements in wearable electronics. It is explicitly noted that this application requires a different processing window than decorative fading or surface activation [72,73,74,75].

3. Wearable and smart textile systems

Laser-activated cotton substrates have been successfully integrated into wearable sensors for motion, pressure and physiological monitoring. In these systems, the laser serves primarily as a surface activation and patterning tool, enabling precise placement and durable adhesion of conductive materials [73,76].

### 5.5. Nanoparticle Immobilisation and Hybrid Composites

Laser-activated cotton surfaces provide a versatile platform for immobilising metal and metal-oxide nanoparticles. Silver nanoparticles, for example, can be anchored through coordination with carboxyl and carbonyl groups formed during oxidation, resulting in durable antibacterial performance [34,77]. Similarly, titanium dioxide and zinc oxide nanoparticles show improved nucleation, distribution and adhesion on laser-treated substrates, leading to enhanced photocatalytic, UV-protective and self-cleaning properties [34,78].

The combination of nanoparticles with laser-induced micro-texturing yields hybrid surfaces in which chemical bonding and mechanical interlocking act synergistically, improving retention of functional particles under bending, rubbing and washing, which is critical for long-term performance.

### 5.6. Polymer Grafting and Advanced Materials Integration

The presence of reactive functional groups on laser-oxidised cotton enables graft polymerisation and improved adhesion of a wide range of polymeric systems. Conductive polymers, hydrogels, and stimuli-responsive materials can be grafted onto the activated surface, opening routes towards sensor-integrated and adaptive textiles [79]. The use of CO_2_ laser post-processing also opens up opportunities in the production of functional textiles, on which various metals have initially been sprayed, resulting in a material with specific properties, such as electromagnetic, UV and IR radiation shielding, antistatic, antibacterial, hydrophilic, and hydrophobic properties and increasing wear resistance [80]. The micro-textured morphology enhances mechanical interlocking, further stabilising the polymer–fibre interface [81].

Collectively, these post-laser functionalisation strategies demonstrate that CO_2_ laser processing is not merely a decorative finishing tool but a flexible and efficient platform for engineering advanced textile surfaces.

## 6. Sustainability and Digital Integration

One of the strongest motivations for adopting CO_2_ laser technologies in textile finishing is the potential for substantial improvements in environmental performance. Conventional denim processing is among the most resource-intensive operations in the textile industry, with high consumption of water, chemicals and energy and the generation of large volumes of effluent requiring complex treatment [82,83,84,85]. By contrast, laser finishing is a dry process that eliminates the need for water-based bleaching and abrading baths and can significantly simplify production lines [86,87].

The results of comparative studies indicate that replacing permanganate spraying and stone washing with CO_2_ laser fading can reduce water usage by more than an order of magnitude while simultaneously lowering chemical consumption and sludge generation [24]. The process also reduces energy demand by eliminating the heating of large baths and repeated drying cycles. In addition, the laser system itself consumes electrical power, which is applied locally and intermittently, and overall energy balances are favourable when complete process chains are considered.

Waste minimisation is another important aspect. Laser fading generates no solid or liquid process waste, with the exception of minor dye volatilisation. The ability to re-dye or functionalise previously processed areas supports product life extension and circular economy strategies by enabling repair, refurbishment and repurposing of garments [36,84,88]. Additionally, improved stability of functional finishes on laser-activated substrates reduces the need for reapplication and decreases losses during use and laundering.

From a production perspective, CO_2_ laser systems are highly compatible with digital manufacturing and Industry 4.0 concepts. They can be integrated with computer-aided design (CAD) systems, enabling rapid implementation of complex patterns and mass customisation [89]. Process parameters can be stored, monitored and optimised in real time, with inline sensing and feedback control offering the possibility of tightly controlled and reproducible outcomes [90]. This digital connectivity facilitates data collection at scale, which is essential for subsequent application of machine learning techniques to predict process–structure–property relationships.

Concurrently, the introduction of laser systems involves substantial capital investment and requires operator training and process optimisation. Successful deployment at industrial scale depends on rigorous parameter mapping, robust hardware and clear demonstration of economic and environmental benefits relative to incumbent technologies.

## 7. Challenges and Future Directions

Despite considerable progress in both scientific understanding and industrial implementation, several challenges continue to limit the full exploitation of CO_2_ laser processing in textile finishing.

### 7.1. Coverage of Emerging Directions in the Last Five Years

A recurring concern in the recent textile laser literature is that processing workflows increasingly combine multiple physical surface activation methods to expand the attainable property space and to improve robustness. In cotton and cotton-rich textiles, the most relevant emerging directions include (i) hybrid surface activation routes that combine laser processing with plasma or other physical pre-treatments, and (ii) CO_2_-laser processing of newer cotton-based material classes, including regenerated cotton and functional blended cotton (for example, cotton/linen or cotton/recycled polyester) that are becoming more prevalent in circular-economy manufacturing.

Within the strict CO_2_-laser + cotton focus of this review, the indexed literature that simultaneously satisfies (a) CO_2_ laser irradiation as the primary energy source, (b) cotton/cellulose as the dominant interacting substrate, and (c) sufficiently reported processing parameters remains concentrated on conventional cotton and denim substrates. Hybrid routes are represented mainly through adjacent studies that use plasma as a complementary activation pathway for cellulose-containing textiles, which motivates transfer of comparative concepts (for example, depth-resolved surface chemistry) but does not yet provide a sufficiently dense CO_2_-laser hybrid dataset for systematic collation. Similarly, while cotton/polyester blends are covered in the conventional literature, the specific case of regenerated cotton and cotton blends that incorporate recycled polymers is still under-represented in the CO_2_-laser processing literature base.

Accordingly, we explicitly identify hybrid CO_2_-laser/plasma and CO_2_-laser/ultrasound routes, as well as regenerated and circular-economy cotton blends, as priority areas for systematic future reviews and benchmarking campaigns. A rigorous synthesis in this direction will require PRISMA-style systematic searches, explicit exclusion criteria, and bias checks (for example, database and language coverage and the regional distribution of contributing laboratories) to ensure that conclusions generalise beyond the well-studied denim-fading use case.

### 7.2. Comparative Framework for Quantifying Surface Chemistry and Oxidation Depth

Additionally, many studies report laser-induced oxidation using surface-sensitive spectroscopy; the field still lacks a shared comparative framework that clarifies what each characterisation route measures (chemical functionality versus depth distribution), which limitations constrain quantitative interpretation, and which methods are best aligned with specific application requirements. Table 4 provides a practical comparison of the most relevant techniques and the type of evidence they deliver for CO_2_-laser-modified cotton surfaces.

### 7.3. Differentiated Requirements Across Application Scenarios

The same CO_2_ laser parameter window cannot be assumed to satisfy all cotton-based textile applications. Apparel and denim finishing prioritise colour control, pattern sharpness, fabric hand and mechanical integrity; therefore, the acceptable processing window is typically constrained to regimes dominated by dye decomposition and limited oxidation, with strict avoidance of yellowing, charring and strength loss. In contrast, industrial and technical cotton fabrics that target coating adhesion, barrier performance, antimicrobial functionality or electronic integration may intentionally operate in regimes with stronger oxidation and micro-texturing to maximise surface energy and anchoring capacity, provided that durability targets are met.

This application dependence should be reflected in both reporting and process qualification. In practice, apparel workflows should report, at minimum, colour metrics (L*, a*, b*, K/S, or ΔE), handle-related indicators and tensile/tear performance alongside key laser settings, whereas technical and industrial workflows should additionally report adhesion metrics, wetting/uptake behaviour and functional durability under standardised wash/abrasion ageing. Aligning characterisation with the intended use case reduces the risk of over-generalising results from denim fading to function-driven processing routes.

### 7.4. Prioritised Implementation Roadmap: Short-Term vs. Long-Term Directions

To operationalise the future directions proposed in this review, we distinguish near-term actions that are feasible with current laboratory and industrial capabilities from longer-term research that depends on deeper modelling and data infrastructure.

Short-term (0–2 years): (i) adopt a minimum reporting checklist for CO_2_ laser textile studies (power/fluence, speed, resolution/overlap, spot size, focus condition, number of passes, fabric composition, areal density, moisture state); (ii) publish parameter–response maps for representative fabrics and machines; (iii) implement standard durability protocols for functionalised surfaces (washing, abrasion, UV exposure) so that stability claims are comparable across studies.

Medium-term (2–5 years): (i) expand depth-resolved characterisation of oxidation and ablation using a harmonised subset of methods (Table 4) and round-robin interlaboratory testing; (ii) develop physics-informed thermal and oxidative models that link delivered thermal budget to oxidation depth and micro-ablation thresholds across fabric architectures; (iii) extend benchmarking to regenerated cotton and cotton blends that incorporate recycled polymer components.

Long-term (5+ years): (i) create open, machine-agnostic datasets that enable robust machine learning models for predicting process–structure–property outcomes; (ii) develop data-driven digital twins and closed-loop control for in-line optimisation; (iii) evaluate hybrid CO_2_-laser processing routes (for example, laser + plasma) under unified metrics to quantify whether hybridisation improves robustness or expands the attainable functional design space relative to single-step laser processing.

A first challenge is the lack of standardised reporting frameworks. Researchers often employ different combinations of fluence, power, scanning speed, DPI, beam diameter and focusing conditions, sometimes without complete documentation [91,92,93]. This variability complicates comparison between investigations and hinders the definition of broadly applicable processing windows. The development of a consistent parameter notation and minimum reporting requirements would greatly facilitate knowledge transfer and meta-analysis. The minimum reporting checklist proposed in Section 7.4 is intended as an actionable starting point for cross-study comparability.

A second challenge lies in the quantitative modelling of thermal behaviour and oxidation depth. While qualitative descriptions of photothermal mechanisms are well established [91,94], detailed models that couple laser energy deposition, heat conduction, chemical decomposition and morphological evolution remain relatively scarce. Depth-resolved analyses of oxidation and ablation are still limited, and most spectroscopic data provide only averaged surface information. Coupling advanced characterisation techniques with multi-physics simulations offers a promising route towards predictive models capable of guiding process design [91,95]. A consistent comparison of characterisation routes, such as that summarised in Table 4, is necessary to make oxidation-depth claims interpretable and transferable across laboratories and machine platforms.

A third area requiring further work is the long-term stability and durability of functionalised surfaces. Many studies report initial improvements in dyeability, coating adhesion and nanoparticle retention on laser-activated substrates [91,96], but systematic evaluations under realistic conditions of wear, washing, UV exposure and mechanical stress are less common. Standardised testing protocols and long-term ageing studies are needed to ensure that functional benefits persist throughout the product life cycle. From an application viewpoint, durability should be assessed against use-case-relevant ageing protocols (washing, abrasion and, where applicable, UV exposure), rather than being inferred from initial post-treatment performance alone.

Finally, the increasing complexity of parameter–material–function relationships calls for data-driven approaches. The combination of multi-dimensional process spaces, varied fabric structures and diverse functional targets yields a challenging landscape that is difficult to map by means of conventional trial-and-error methods. Machine learning and other data analytics tools have begun to be applied to the predictive modelling of laser–material interactions, but their use in the specific context of textile finishing is still limited. Progress in this direction depends on the availability of curated datasets, consistent evaluation metrics and closer integration between experimental and computational work.

The development of new non-woven materials also opens up new opportunities for the application of laser processing to modify and improve their properties [97,98,99,100].

Addressing these challenges will be essential for the transition of CO_2_ laser textile technologies from primarily decorative denim finishing tools to robust, multifunctional and application-driven platforms for material modification.

## 8. Conclusions

CO_2_ laser processing of cotton and denim textiles is a mature yet dynamically evolving technique that offers precise, digitally controllable and environmentally favourable alternatives to conventional wet finishing. The interaction is governed by a sequence of photothermal mechanisms in which cellulose strongly absorbs 10.6 µm radiation, leading to local heating, dye decomposition, surface oxidation and, at higher thermal budgets, micro-ablation. The consolidated evidence supports that outcomes can be made predictable and transferable when process variables are reported in a comparable manner and when parameter–response maps are constructed for a given machine–fabric combination; by contrast, incomplete reporting and fabric-to-fabric variability remain the primary barriers to generalised predictability.

The oxidised and micro-textured surfaces generated by CO_2_ laser treatment exhibit enhanced polarity, wettability and anchoring capacity, enabling improved dyeability and adhesion of functional coatings and nanoparticles. However, the functional relevance of these surface states depends on the application scenario: apparel finishing requires tight control of colour and hand with minimal structural damage, whereas technical and industrial textiles may deliberately prioritise activation depth and micro-texturing to maximise adhesion and durability. Therefore, stability claims should be supported by use-case-relevant ageing protocols rather than by initial post-treatment performance alone.

From a sustainability perspective, CO_2_ laser finishing is intrinsically water-free at the treatment step and can replace or reduce high-effluent operations such as chemical bleaching and abrasive wet washes. Comparative studies reported in the literature indicate order-of-magnitude reductions in water usage when laser fading substitutes selected conventional denim finishing steps, alongside reductions in chemical consumption and sludge generation. Nevertheless, quantitative cross-study integration remains constrained by heterogeneous reporting of process settings, functional metrics and durability tests, highlighting the need for standardised datasets and benchmarking.

Based on the current technical maturity, we prioritise the following implementation path: in the short term, standardise minimum parameter reporting, publish parameter–response maps and adopt comparable durability protocols; in the medium term, expand depth-resolved characterisation and physics-informed thermal and oxidative models; and in the long term, develop open datasets, data-driven digital twins and validated hybrid processing routes. Collectively, these steps provide a concrete route toward scalable, evidence-based and sustainability-oriented CO_2_ laser processing of cotton-based textiles.

## Figures and Tables

**Table 1:** Qualitative correspondence between the simplified phenomena depicted in Figure 1 and the underlying mechanisms in the context of CO_2_ laser processing of cotton fabrics.

Phenomenon	Mechanism in Laser + Cotton Context
“Thermal ablation: remove dye + some fibre”	Laser energy heats and vaporises or decomposes indigo dye on the fibre surface and sometimes removes pieces of fibre.
“Oxidation: small graphic of oxygen or ‘O_2_’ + heat symbol”	Ambient oxygen + heat cause chemical oxidation of cellulose (OH → carbonyl/carboxyl), creating new functional groups.
“Pore formation: little holes/micro-pits on fibre surface”	Localised heating/decomposition leads to etching, cracks, and micro-voids visible under an SEM.
“Thermal removal of indigo dye”	As under ablation, indigo dye molecules decompose or are removed, lowering colour yield (K/S).
“Formation of micro-pores/porosity”	Etching from repeated laser spots causes a porous, sponge-like structure, increasing surface area.
“Thermal oxidation → yellowing”	Oxidative degradation leaves charring or oxidised residues, which visually appear yellow or green (with residual blue dye).

**Table 2:** Representative grouping of process parameters relevant to CO_2_ laser treatment of cotton and denim fabrics.

Group	Parameters
Laser settings	Power, pulse duration, speed, wavelength, scanning pitch/overlap
Optical configuration	Beam spot diameter, focal length, defocusing
Scanning strategy	Hatch pattern, direction, number of passes
Material characteristics	Fibre composition, weave/knit, finishing chemicals, humidity

**Table 3:** Typical qualitative correlation between observed effects in CO_2_ laser-treated cotton fabrics and the dominant underlying mechanisms.

Observed Effect	Mechanism
Whitening/fading	Oxidation of indigo
Brown/black shades	Carbonisation of cellulose
Roughening	Micro-ablation of surface fibres
Decrease in tensile strength	Thermal degradation
Increased hydrophilicity	Micro-fibrillation and oxygen-containing groups
Increased surface conductivity (rare)	Carbonised surface under high fluence

**Table 4:** Comparative overview of characterisation routes for CO2 laser-induced surface modification and oxidation depth in cotton-based textiles.

Technique	Primary Information Returned	Depth Sensitivity/Spatial Resolution	Strengths	Limitations and Best-Fit Scenarios
ATR-FTIR/FTIR mapping	Functional groups (C=O, O–C=O), oxidation fingerprints	Near-surface (typically micrometre-scale); mapping at 10–100 µm scale	Rapid, accessible, and directly links to chemical functionality	Limited depth resolution; signal averages over heterogeneous yarn/fibre topography; best for screening and comparative trend analysis
XPS	Surface elemental composition and bonding states; O/C ratio; carbonyl/carboxyl components	Top few nanometres; lateral resolution typically tens of µm	Highly surface-sensitive and quantitative for chemistry	Does not resolve depth without profiling; requires vacuum and careful charge compensation; best for verifying true surface activation
XPS depth profiling (sputter-based)	Approximate depth distribution of oxidised layer	Depth-resolved but method-dependent; risk of artefacts in polymers	Provides the most direct route to oxidation-depth estimates when carefully controlled	Sputter damage/reduction can distort cellulose chemistry; requires method standardisation; best for research-level mechanistic studies and benchmarking
SIMS/ToF-SIMS	Depth and lateral distribution of characteristic fragments and contaminants	High surface sensitivity; nanoscale depth; µm-scale lateral mapping	Rich chemical fingerprint; can detect low-concentration species	Quantification is non-trivial; instrumentation access is limited; best for resolving heterogeneous oxidation and finish residues
Raman/micro-Raman mapping	Carbonisation signatures, structural disorder, dye residues; spatial mapping	Micrometre-scale lateral; optical penetration depends on the sample	Non-destructive mapping complements FTIR for carbonisation and dye chemistry	Fluorescence and weak Raman in cellulose can limit; best for identifying carbonised regimes and pattern uniformity
SEM (optionally with EDS)	Surface morphology: fibrillation, pits, cracks; micro-ablation features	High spatial resolution (nm–µm) but mainly morphological	Direct visualisation of micro-ablation and texture evolution	Chemical interpretation indirect; sample charging/prep considerations; best for linking process settings to micro-texture and damage thresholds
AFM/optical profilometry	Topography and roughness metrics (Ra, Rq); texture anisotropy	nm–µm vertical resolution; limited scan area (AFM)	Quantifies micro-texture that drives adhesion/wettability	Limited representativeness on woven fabrics; best for controlled substrates or local features
Wettability/uptake tests (contact angle, wicking) + durability tests	Effective surface energy and capillary transport; functional stability under washing/abrasion	Bulk-integrated response across the yarn network	Application-relevant, low cost; links directly to performance targets	Not mechanism-specific; must be paired with spectroscopy/microscopy; best for qualifying parameter windows for apparel vs. technical/industrial textiles

## Data Availability

No new data were created or analysed in this study. Data sharing is not applicable to this article.
